# Draft genome sequence of *Streptomyces noursei* strain DS30.6, isolated from forest soil in Vietnam

**DOI:** 10.1128/mra.00782-24

**Published:** 2024-11-08

**Authors:** Binh Thanh Le, Ngoc Minh Truong, Quang Dinh Vo, Trang Thi Phuong Phan

**Affiliations:** 1 Faculty of Biology and Biotechnology, University of Science, Vietnam National University Ho Chi Minh City, Ho Chi Minh, Vietnam; 2 Vietnam National University, Ho Chi Minh, Vietnam; 3 National Center for Technological Progress–HCM Branch, Ho Chi Minh, Vietnam; DOE Joint Genome Institute, Berkeley, California, USA

**Keywords:** draft genome, *Streptomyces*, plant diseases, *Ralstonia*

## Abstract

*Streptomyces noursei* strain DS30.6 was chosen to be resistant to *Ralstonia pseudosolanacearum* T2C-Rasto after being isolated from Vietnamese forest soil. The draft genome of this strain was reported to be 8,863,720 bp, with an average guanine-cytosine content of 72.67%.

## ANNOUNCEMENT

*Ralstonia solanacearum* is the causative agent of severe bacterial wilt disease in many crops (1). We previously determined that the *Ralstonia pseudosolanacearum* T2C-Rasto caused bacterial wilt in cucumbers in An Giang, Vietnam (2). Here, by isolating and selecting *Streptomyces* strains resistant to T2C-Rasto, we report the draft genome sequence of *Streptomyces noursei* DS30.6, which was found to have an antagonistic effect against T2C-Rasto. The biological control potential of *S. noursei* was investigated against *Aspergillus niger* and *Pseudopestalotiopsis camelliae-sinensis* (3, 4).

*Streptomyces* sp. was isolated from diverse soil samples collected in Vietnam’s Mekong Delta. The isolates were cultured on Gause-I medium at 28°C for a period of 7–14 days. These *Streptomyces* strains were screened for antagonistic activity against T2C-Rasto by using the direct agar bar method. In this method, 7-mm wells were created on T2C-Rasto plates, and agar bars of *Streptomyces* sp. with the same diameter were inserted into the wells. After incubation at 28°C for 72 hours, six *Streptomyces* isolates with the highest inhibitory activity, as indicated by the largest inhibitory zones, were selected for further indirect plate diffusion examination. We used 1 mL of *Streptomyces* culture (OD600 = 1) without cells, which was added into the wells (7 mm in diameter) of T2C-Rasto plates and incubated at 28°C for 48 hours. The diameter of the inhibition zone was calculated by subtracting the well diameter from the antagonistic ring diameter. The strain showing the strongest resistance was identified as *Streptomyces noursei* DS30.6 from the forested area, through investigation of the 16S rRNA gene sequence using Sanger sequencing. The DS30.6 sample was collected in the morning from Bay Nui Mountain, An Giang, Vietnam. A single isolated colony of DS30.6 was selected for genome sequencing. Genomic DNA was extracted using a DNA mini kit (Qiagen, Germany) and sequenced using the Illumina Miniseq platform at KTest Co., Ltd., with the NEBNext Ultra II DNA Library Prep Kit 2 × 150 bp. Low-quality reads were filtered using Trimmomatic 0.36 (5) with default settings, resulting in a total of 2,350,115 paired reads, and the filtered reads were assembled using Unicycler 0.4.4 with default metrics (6). The genome annotation was carried out by National Center for Biotechnology Information (NCBI) Prokaryotic Genome Annotation Pipeline (PGAP) with default parameters (7). CheckM (v.1.2.2) was incorporated in the PGAP of the NCBI genome and was used to assess the quality of the genome.

The draft genome of DS30.6, estimated to be 8,863,720 bp, was derived from 142 contigs, with the largest contig being 685,059 bp. It features a guanine-cytosine content of 72.67%, an N_50_ value of 183,934 bp, and an estimated completeness of 99.79%. The genome contains 7,844 predicted genes, 7,564 coding genes, 216 pseudogenes, 74 RNA genes, 68 tRNA genes, and 3 rRNA genes. Anti-SMASH (v.6.0.1) discovered 34 gene clusters involved in the production of antibacterial compounds (8).

## Data Availability

The draft genome sequence of *Streptomyces noursei* DS30.6 has been deposited in the National Center for Biotechnology Information GenBank under accession number JAJUFB000000000 and Sequence Read Archive number SRR30500778 with BioProject number PRJNA789597 and BioSample number SAMN43434456.
